# A Case Study of 21st Century Cognitive, Social and Emotional Competencies Using Online-Learning

**DOI:** 10.3390/jintelligence11060116

**Published:** 2023-06-09

**Authors:** Haïfat Maoulida, Manisha Madhukar, Macarena-Paz Celume

**Affiliations:** 1 Laboratoire de Psychologie et d’Ergonomie Appliquées (LaPEA, UMR 7708T), Institut de Psychologie, Université de Paris Cité, 92100 Boulogne-Billancourt, France; 2 Beyond Education, Learning Planet Institute (LPI, ex CRI), 75004 Paris, France; manisha@beyondeducation.tech; 3 Centre de Recherche en Psychologie de la Connaissance, du Langage et de l’Émotion (Centre PsyCLE, EA 3273), Institut Créativité et Innovations, Université Aix-Marseille, 13385 Marseille, France; mp.celume@cri-paris.org

**Keywords:** socio-emotional skills, 21st century competencies, online program, educational program, creativity, critical thinking, mindfulness, resilience, metacognition

## Abstract

Based on the conceptualisation of the 21st Century Competencies Framework from the Center for Curriculum Redesign (CCR) we developed an online program to enable school-age students to increase their level on several social-emotional competencies. BE organized is a program that aims to help students to better organize themselves to be more efficient in today’s and tomorrow’s world. To do so, 12 individual sessions were designed to develop 4 out of the twelve 21st century competencies: *Critical Thinking*, *Mindfulness*, *Resilience* and *Metacognition*; collective sessions (action lab) to develop others such as *Creativity*. We used a mixed methodology, i.e., quantitative (two questionnaires) and qualitative (reflective questions) evaluation to test whether the targeted competencies have been developed during this program. Preliminary results (since it involves only a small number of participants, *n =* 27) partially confirm our hypotheses. Both qualitative and quantitative data show a development of critical thinking; the cross-sectional results are more mixed for the other three targeted competencies. Moreover, some other competencies, such as *Creativity* and *Growth Mindset,* seem to be developed during this program. However, it is difficult to determine whether it is the group and/or individual sessions that are responsible for these non-targeted competencies development. These results will be discussed in relation to the youth literature on 21st century competency and the broader literature on socio-emotional learning (SEL) and/or emotional intelligence (EI).

## 1. Introduction

### 1.1. 21st Century Cognitive, Social and Emotional Competencies: An Anchor in Emotional Intelligence (EI) Theory?

In recent years, new models have proposed to group essentially non-academic socio-emotional competencies into more integrative models that combine traditional or classical knowledge (e.g., STEM) with cognitive, behavioural, social and emotional competencies under the term ‘21st century competencies’ (Soffel 2016). The first definition of these competencies is the set of abilities that are indispensable for an individual to evolve in the current socio-economic world (González-Salamanca et al. 2020; UNESCO 2017). Yet, there is no clear and unique definition of ‘21st Century Skills’ (Joynes et al. 2019). Joynes et al. (2019) cite the term ‘21st Century Skills’—or ‘21st century competencies’—as ‘an overarching concept for the knowledge, skills and dispositions that citizens need to be able to contribute to societal knowledge. The ambiguity in terminology and definitions hinders the ways in which we think about and teach such skills. In particular, this concept is often associated or even confused with emotional intelligence. Emotional intelligence (EI) can be synthetically defined as the ability to process emotional information efficiently and accurately (Mayer and Salovey 1997). It also refers to four main skills: perceiving, understanding, assimilating or integrating, and managing emotions (Mayer and Cobb 2000). Many models of emotional intelligence exist. Drigas and Papoutsi (2018, 2023) propose, like their model of general intelligence (Drigas and Pappas 2017), a pyramidal vision of intelligence in 9 layers that a human must cross to reach the higher, ultimate level, which is emotional unity (Drigas and Papoutsi 2018, 2023). Nevertheless, when it comes to adopting a policy, particularly an educational one, to develop emotional intelligence, it is more important to start from the development of socio-emotional competencies (Mayer and Cobb 2000). Socio-emotional competencies can be defined as the capacities necessary to evolve in a social world (Elias 2003). According to The Collaborative for Academic, Social, and Emotional Learning (CASEL), these competencies encompass the ability to (1) know oneself and others, able to identify emotions—take responsibility—recognise strengths; (2) make responsible decisions—the ability to manage emotions—understand situations—set and plan goals and objectives—creatively solve problems; (3) care, showing empathy—respecting others—embracing diversity; (4) knowing how to act, namely, communicating effectively—knowing how to build relationships—negotiating fairly—seeking help—acting ethically (Elias 2003). Similar to emotional intelligence models (e.g., Bar-On 2006; Mayer and Salovey 1997; Petrides et al. 2006), there are multiple socio-emotional learning (SEL) models, but policies from one country to another use SEL curriculum development to promote students’ EI (Wood 2020); developing their ability to demonstrate self-management, self- and social awareness, and ethical and responsible decision making (Dusenbury and Weissberg 2017). Indeed, SEL or EI research has demonstrated positive effects on different aspects of personal, professional and school life.

### 1.2. Beneficial Effects of Developing These Competencies

SEL studies have demonstrated positive impacts on school performance, well-being, prosocial behaviour, and others (Wood 2020). Indeed, students that benefited from an SEL program showed significantly more positive outcomes, such as lower levels of behaviour problems or emotional distress and higher positive social behaviour, and higher academic performance, compared to control students (Mahoney et al. 2018). So these competencies seem to be important for today’s world but are especially important for the potential they have to build a better, more sustainable world (González-Salamanca et al. 2020; UNESCO 2017). Nowadays, our societies are evolving rapidly, implying that the jobs of tomorrow, the socio-economic problems, and the technologies we interact with will no longer exist (World Economic Forum 2020). There appears to be a consensus regarding the need to develop and measure non-academic competencies that offer ways to better equip students and new generations to adapt to our fast-changing world. There is some evidence about the effectiveness of developing these competencies at school, particularly through EI models, in order to increase and enhance students’ prosocial behaviours (Wang et al. 2021), academic performance (Poropat 2009; Sánchez-Álvarez et al. 2020), and diverse positive outcomes (e.g., Lipnevich et al. 2016; Roberts et al. 2007). Indeed, an important element for the implementation of such programs is that they are based on a strong scientific and theoretical model (Lamboy et al. 2022a, 2022b), hence our motivation to select a program model from the scientific literature in the fields of education and/or psychology.

### 1.3. Four-Dimensional Education from the Center for Curriculum Redesign: A 21st Century Competencies Model to Develop and Assess a Program

It is important for us to look for the most comprehensive model to develop evidence-based programs since the perception of the theoretical model has an impact on the content and effectiveness of a program (Wood 2020). Several models have been developed to determine what these competencies are. We use the Center for Curriculum Redesign (CCR; Fadel et al. 2015). Researchers worked on the creation of several frameworks based on the theories of EI to target nonacademic competencies that are needed to succeed in academic and/or professional life. In their 4D model, the CCR divides 21st century competencies into the following four dimensions: the first dimension is traditional knowledge (mathematics, literacy, STEM, etc., Bialik and Fadel 2018). The second dimension is skills, which refers to the effective and appropriate use of the knowledge that an individual possesses, acquires or seeks to acquire (Bialik et al. 2015a). Generally, these skills are grouped as “4Cs” for Creativity, Critical Thinking, Communication and Collaboration (The 4Cs within the CCR model are different from the famous four C model of Creativity proposed by Kaufman and Beghetto (2009)). The penultimate dimension brings together a set of character traits that refer to the way a person engages and behaves in a constantly changing Society (Bialik et al. 2015b). These are Leadership, Ethics, Resilience, Courage, Curiosity, and Mindfulness (Fadel et al. 2015). The last dimension is Meta-learning that brings together all the abilities that allow a student to have a reflection on their thinking process (metacognition) and to perceive that they will be able to learn throughout their life and that their intelligence is not fixed but can grow, known as a growth mindset (Bialik and Fadel 2015). All these dimensions interact with each other (Fadel et al. 2015). Nevertheless, these competencies remain difficult to measure, and no universal assessment has yet been adopted (Ananiadou and Claro 2009; Bialik et al. 2016). Yet how can we determine that students are developing competencies through these programs if there is no tool to do so? Therefore, in order to assess a program, it seems essential to have a tool or other means of assessing these competencies (Bialik et al. 2016; Celume and Maoulida 2022a). Thus, we need to think of 21st century competency development programs that also are able to assess them in a systematic way (Bialik et al. 2016; Celume and Maoulida 2022b; Slavin 2008). Indeed, there is a disconnection between the education system and the competencies needed in our uncertain job market (World Economic Forum 2021a, 2021b). To bridge the gap between what is expected from the job market and the competencies students possess, this program aims to bridge a part of this gap by using an evidence-based approach (see Zins et al. (2007) or Wood (2020)) within an online, action-research program that strengthens 21st century competencies. The purpose of this study is to demonstrate the effectiveness of a program to develop these 21st century competencies as an external institution to English-speaking students from around the world. Indeed, while there are programs that develop SELs, the program proposed here goes beyond what is defined as SELs, namely, 21st century cognitive-socio-emotional competencies, both academic and non-academic, in an online program that aims to be as universal as possible.

### 1.4. Content of 21st Century Competencies Development Program

There are some efforts to include SEL inside traditional classrooms and teaching. For example, some recent works and metanalyses (e.g., Fajrina et al. 2020) have shown that the STEM learning (i.e., identified as “classical knowledge” learned at school) approach is a global movement of educational practice that incorporates different models of integration to improve students’ 21st century competencies, specifically the famous “four Cs”: Critical Thinking, Creativity, Collaboration and Communication. In addition to the fact that these practices are not generalized nor homogeneous in all schools across our societies, STEM would not allow the development of all 21st century skills (see below the list of competencies that we put in this group). Moreover, most of the SEL studies have taken place in US schools or in the European context (Cristóvão et al. 2017). Whether in Europe or in the US, there are diverse programs proposed (Cristóvão et al. 2017; Torrente et al. 2015). Yet, there does not seem to be a consensus on the content of cognitive-socio-emotional competency development programs, particularly as the cultural context may have an implication (Wood 2020). Nevertheless, it seems important to achieve standardization in the programs, meaning a program whose content can be utilized (with minor adjustments) regardless of the context in which it is implemented (Wood 2020).

### 1.5. Developing 21st Century Competencies Online

In a post-COVID-19 world, this unprecedented health crisis has led the world of education to rethink its practices to invest in online networks (Katzman and Stanton 2020; McQuirter 2020). Even though the need to develop non-academic competencies is apparent, there are some challenges in terms of online cognitive, social, and emotional competencies development. Cinque (2017) posits that MOOCs (Massive Online Open Courses) require individual learners to self-regulate their learning, determining when and how they engage. Additionally, MOOCs attract a diverse range of learners, each with different motivations and prior experience (Cinque 2017). Cinque (2017) also adds that it might seem quite challenging to develop non-academic skills through online resources since these skills are mainly behavioural. On the other hand, logistical challenges such as a lack of effective teacher training (Sarwanto et al. 2021) or financial or time investment by schools in these types of programs highlight the need for online courses. Moreover, Onofrei (2015) suggests that teacher beliefs—that their IT skills are frequently inferior to students’ IT skills—complicates their development and warns that when technology is not integrated into the academic arena, students may not understand the extent to which technologies can support learning. This may hinder the development of new learning styles and their ability to adapt and cope with the changing world (Onofrei 2015). Considering the challenges mentioned in the literature as well as the need for digitization of competencies-building, it appears that an online program may be useful to mitigate logistical hindrances such as ineffective teacher training and heavy investment by schools. Therefore, it seemed essential to develop this program online, especially since SEL curricula have a positive effect, whether in the traditional classroom at school or within online asynchronous learning platforms (Durlak et al. 2011).

An online program in the most widely spoken language in the world (i.e., English) has the advantage of not only reaching more students (thus standardizing the learning offered) but also reaching students less targeted by these programs outside of the US and Europe; helping to address educational disparities (Katzman and Stanton 2020).

In addition, developing these competencies online improves digital literacy (Katzman and Stanton 2020). Thus, a virtuous circle exists between learning these competencies online and better online skills for learning. The question was as follows: “How do we develop these competencies?”. The recommendations in this area are in favour of using evidence-based programs (CASEL 2020; Kamei and Harriott 2020). These programs should use clear instructions in terms of SEL and integrate with pre-existing academic practices (Kamei and Harriott 2020). In the first case, instructions should be explicit within the lessons on each of the competencies covered with a scientific base to build this lesson (Kamei and Harriott 2020). This is the path we adopt by using a theoretical framework that defines 21st century competencies and by implementing an evaluation of this program through the use of quantitative (measurement tools measuring these competencies) and qualitative (a content analysis of reflective responses and interviews) methods.

### 1.6. Construction of the Online 21st Century Competencies Development Program: Be Organized

BE Organized is a program created by Beyond Education based on the Four-Dimension Educational Model from the Center for Curriculum Redesign (CCR, Fadel et al. 2015). Each program proposed by Beyond Education targets 3–5 core competencies to develop out of the twelve possible cognitive-socio-emotional competencies. Be Organized is designed to address an issue that seems to come up often: self-management. Self-management can be defined as the ability of an individual to regulate their tasks and activities to achieve a desired goal. In many interventions on self-management, students learn to self-evaluate, monitor, and reinforce academic skills (Langberg et al. 2008). Among the cognitive-socio-emotional competency groups proposed by the SEL, self-management also refers to the ability to regulate one’s emotions, thoughts, and behaviours effectively in different situations and to achieve goals and aspirations (Kamei and Harriott 2020). According to authors in the field, a set of five broad families of interventions can be identified: self-instruction, self-monitoring, self-assessment, strategy teaching, and goal setting (Mooney et al. 2005). Indeed, teachers often assume that students, by the time they reach middle school, are developed enough to juggle multiple assignments and lessons on their own, are able to plan and organize projects on their own (or collectively), and can regulate their time and behaviour without assistance and guidance (Bakunas and Holley 2004). Viewing organizational competencies as part of a developmental learning process helps teachers understand that students may need support and instruction before they can take responsibility for their learning (Bakunas and Holley 2004). Furthermore, the issue of organizational problems has often been raised with time-intensive students (e.g., students with emotional and behavioural disorders (Mooney et al. 2005), students with attention deficit disorder and hyperactivity (Langberg et al. 2008), students with autism (Carr et al. 2014), etc.), yet we can hypothesize that the previously mentioned study encourages the view that self-organization and management is a competency that needs to be worked on in all students. Through this online program, the idea is to develop students’ abilities outside school time. If we look closely, the main components of the student’s self-organization of educational activity appear to be goal setting and motivation (Agranovich et al. 2019). Therefore, we mapped these elements of self-organization with the work on the 12 cognitive-socio-emotional competencies taken from the 4D model of the CCR (Fadel et al. 2015) to determine the four competencies of the 4D that matched the most with this main component of self-organization. The aim was to help students develop a concise list of competencies that would make them more self-organized in their everyday home and school life.

### 1.7. Core Competencies Developed by the BE Organized Program

BE Organized aims to help students to better manage their time and energy within an environment full of information and distractions. The purpose of this program is to give them a way to know how to get things performed more efficiently and effectively while finding a sense of balance in the current digital, fast-changing society. This program spans a duration of 8 weeks, containing 12 individual sessions aimed at developing four core competencies: Critical Thinking (Skills), Metacognition (Meta-learning), Mindfulness (Character) and Resilience (Character); that for us, were the four competencies from the original CCR framework that was the most linked to Self-organization in student activities. Each 30–45-min session targets one sub-competency within the four core competencies. These individual sessions contain videos, activities and games to engage various senses of the students and are intertwined with knowledge about current and contextual information. The 4 core competencies of focus in the individual sessions are defined in the CCR (Bialik et al. 2015a, 2015b; Bialik and Fadel 2015; Fadel et al. 2015) as follows:

- Critical Thinking: This skill refers to the mental processes, strategies, and representations people use to solve problems, make decisions, and learn new concepts. Critically evaluating information and claims the individual is confronted with. Using logic and reasoning to identify the strengths and weaknesses of alternative solutions, conclusions or approaches to problems;
- Mindfulness: This character competency will allow an individual to be aware of multiple perspectives, being present in a state of conscious awareness of oneself, their body, thoughts, emotions and environment. In this state, individuals develop and adopt an openness to novelty in which they actively construct categories and distinctions;
- Resilience: This character competency makes an individual able to deal appropriately with the ambiguity, changes, and challenges that different perspectives and experiences can present and to maintain one’s identity and/or develop personally;
- Metacognition: This meta-learning competency allows an individual to be able to recognize one’s knowledge, skills, attitudes/values and way of learning. It also makes a person able to set goals and adapt learning strategies and based on outcomes.

### 1.8. BE Organized Presentation

More specifically, each session is constructed around one of the target sub-competencies (see Table 1). These sub-competencies are presented as the learning objectives of the session and shared with students. In the introductory part of the session, a real-life example is used to illustrate and contextualize the sub-competency, and the main learning objective is announced, how to get there and the underlying learning objectives. Afterwards, activities using a variety of learning and communication mediums are used. Students progressively experience competencies through exercises. The session ends with an optional activity called “individual challenge”, which nudges students to use their learning in contexts outside of the online sessions. This is followed by a “reflection question”, which allows students to gather their thoughts about the session. Finally, a reminder of the session’s objectives is given to show them what they have worked and learned through the session. For example, in one of the sessions of BE Organized, students perform activities to better organize the plethora of information they encounter in their everyday lives. This session targets one of the sub-competencies of Critical Thinking, Identifying, Clarifying and Organizing information. One of the activities, in particular, included the usage of LATCH: Location, Alphabet, Time, Category, or Hierarchy to organize information about a topic that the students selected. LATCH was coined by Richard Saul Wurman, an architect and designer, who provided an “architecture” for information- the way to organize information. The activity is as follows: Be your own information architect: Select one of the five options (Location, Alphabet, Time, Category, or Hierarchy) and organize all the information you have collected about your topic of interest using the selected option.

In addition to the individual sessions that have been discussed in the previous paragraph, students also participate in seven group sessions or “Action Labs”, which is a virtual space for collaborative work on current issues by using the Design For Change methodology (DFC 2015). Owing to these group sessions as well as competency-specific individual sessions, there is value in studying the effectiveness of BE Organized in developing Mindfulness, Critical Thinking, Metacognition and Resilience.

### 1.9. Presentation of This Research

This research aims to study whether, in particular, the individual sessions have made it possible to develop the four central competencies targeted by this program over time (pre- vs. post-program) and compared to other 21st century competencies. Therefore, this study hypothesizes that students will have improved competencies such as Critical Thinking, Metacognition, Mindfulness, and Resilience after completing the BE Organized course, mainly due to individual sessions. We also hypothesize that on some of the remaining eight non-targeted 21st century competencies, which include creativity, we might observe a slight increase owing to the group sessions that develop these competencies. Results will then be discussed regarding several possibilities that the online program offers for the development of 21st century competencies on a large scale. For clarity, and with respect to preliminary results, only part of the analyses conducted will be presented here. Still, both qualitative and quantitative data will be included, and additional analyses will be made available to interested parties. These initial analyses on a small sample will both provide avenues for improvement for the proposed program, as well as room for reflections in the area of 21st century competency development online.

## 2. Materials and Methods

### 2.1. Participants

Two hundred and thirty-seven participants from around the world enrolled in the BE organized program through the Beyond Education website. One hundred and fifty-nine activated their account on the BE platform on Moodle—an open-source learning platform. The activation takes place after user credentials are filled in. Second, participants provide their consent and that of their legal representatives to participate in this program and to the use of their data in this context and for research purposes. The final step is for them to receive a verification email with the information given summarized and their login credentials restored. Of the one hundred and fifty-nine students, one hundred and twenty-seven of them started the program. Finally, there were 27 students from the United Arab Emirates (*n =* 25) and from Slovakia (*n =* 2) who completed the program until the end. The sample was composed of 21 girls (*M =* 13.8 years old, *SD =* 1.76) and 6 boys (*M =* 14.0 years old, *SD =* 2.45) English speakers. The average age of students is 13.82 years (*SD =* 1.88, [11, 18]). The dropout rate is, therefore, 83%, which fits with the dropout range commonly found within the scientific literature on online participation (i.e., from 48 to 99%, Jordan 2015). For quantitative data sources, 27 participants who completed this program were considered. For qualitative data sources, all participants’ responses, including dropouts, were considered (*n =* 93).

### 2.2. Procedure

To analyse the effectiveness of *BE Organized*, 3 instruments were used: Competency Compound Inventory for the 21st Century (CCI-21, Celume and Maoulida 2022b), the Competencies CheckBox Inventory (CBI) and Reflective Question Assessment (RQA).

These 3 instruments were tested within the Beyond Education official platform—Dreamshaper.

1. The CCI-21 questionnaire was conducted prior to and after the completion of the BE Organized programme. Students were restricted from accessing session materials if they had not completed the pre-test (questionnaire);
2. The Reflective Question Assessment (RQA) was conducted after each of the 12 sessions and was mandatory for the students, without which the platform would not provide access to subsequent sessions;
3. The Competencies CheckBox Inventory (CBI) appeared as a part of the “Global satisfaction survey”, which took place at the end of the BE organized program (i.e., when the 12 sessions were completed).

**Figure 1:**
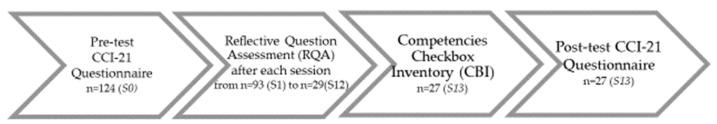
Order of testing instruments in the BE program of 12 sessions (S1 to S12), with two “sessions” 1 week before the start (S0) and right after the end of S12 (S13).

These 3 instruments, based on their placement in the session, were subject to varying numbers of responses. The instruments that were used towards the beginning witnessed the highest number of responses, and the numbers dwindled as the sessions progressed (see Figure 1). The CCI-21 pre-test was taken by 124 students. The RQA of the first session was answered by 93 students, and following progressive reduction of responses, the RQA of the last session was answered by 29 students. The CBI and the CCI-21 post-test were filled in by 27 students.

### 2.3. Materials

#### 2.3.1. CCI-21 Questionnaire

The BE assessment called 21st Competencies Compound Inventory (CCI-21), is a validated questionnaire to measure 21st Century Socio-emotional competencies (Celume and Maoulida 2022b) with 36 questions, ready to be carried out by students through the DreamShaper (DS) platform.

Based on the theoretical framework proposed by the Center for Curriculum Redesign (CCR, Fadel et al. 2015) and some of their recommendations about assessment (Bialik et al. 2016), BE proposed an easy but strong tool to measure 21st century competencies in the school-aged population (10–21 years).

CCI 21, also known as the competency calculator, is a self-reported measure that gathers twelve competencies issued from the four-dimension education framework, Center for Curriculum Redesign. It gives a precise score on each of the twelve measured competencies, as well as a global score on the development of 21st century competencies.

Each of the competencies has a good sensitivity (i.e., no skewness or kurtosis index higher than 1 in absolute value), and most of them (10) have an acceptable to a good internal consistency (α between 0.65 and 0.87), others (2) a little less (α < 0.65). The factorial analyses used principal component by imposing the number of factors expected for each of the 3 components that we analysed in an independent way. We find for the skills component, the 4 expected competencies (λ_1_ = 2.58, λ_2_ = 2.37, λ_3_ = 1.93, λ_4_ = 1.30; 67.98% of cumulative variance), for the component character, saturations of some items raise questions (Mindfulness items particularly), but the 6 components have a saturation higher than 1 on the model (explaining, 66.55% of variance); finally, the 2 components of the dimension meta-learning are about equivalent (λ_1_ = 1.95, λ_2_ = 1.77, 61.92% variance explained; an item which does not saturate on the good component). Finally, the internal reliability of each dimension is good to very good (α_skills_ = 0.85, α_characters_ = 0.88, α_metalearning_ = 0.75). As our sample is small, we consider the results to be satisfactory.

The global score is the sum of the scores obtained by the participant for each of the twelve competencies. This score can be understood as the mean level of students regarding their whole set of competencies for the 21st century. CCI scores can range from 36 to 180. A score of 36 corresponds to a participant having answered 1 (minimum note that a student can self-attribute to the behaviour described) to all questions and thus to the lowest level of 21st century competency. A score of 180 corresponds to a student who has scored 5 (maximum note that a student can self-attribute to the behaviour described) on each item and therefore shows 21st century competency at the highest level.

#### 2.3.2. Competencies CheckBox Inventory (CBI)

Towards the end of the session, a global satisfaction questionnaire is rendered via the BE platform (Dreamshaper). Among numerous other questions spanning general appreciation, program format and individual and collective session ratings, students are also provided with a Competencies Checkbox Inventory (CBI), which is a list of 12 competencies (for details see Appendix A). Students are asked to identify the top 3 competencies that they believe to have developed during the course of the session. The CBI is provided twice to discuss developments in Group and Individual sessions. However, the small sample size of the two scales and especially the fact that they are not Likert scales but choices to be made between options prevented us from running a psychometric analysis. 

#### 2.3.3. Reflective Question Assessment (RQA)

At the end of each of the 12 sessions, students are required to reflect on 2 questions that encapsulate their learnings from the session. The following one question is generic and the same throughout all sessions: “What sticks with you about the session?” while another is specific to the session (for details see Appendix B). The open-ended generic question is aimed at recognising main takeaways from the session, while the open-ended specific question targets competency-specific keywords. The questions are constructed based on the description of the sub-competencies proposed by the CCR (Fadel et al. 2015) knowledge gathered from the competency and the developmental stage of students.

#### 2.3.4. Social Desirability Scale (SDS)

Social Desirability Scale (SDS) is a 13-item, binary (i.e., the following two possibilities to answer to each item: true or false) scale proposed by Crowne and Marlowe (1960) in a long version and validated in a short version by Barger (2002). We slightly changed two words in two items to make it more readable by a young population, as it was originally designed for adults. Half of the items were reversed. This scale was put at the beginning of pre- and postprogram assessment. A person can score between 0 and 13; the higher the score, the more a person is willing, consciously or not, to present themselves in a positive suitable way, which is accepted, favoured and appreciated within the society a person lives in (α = 0.70).

### 2.4. Data Analysis

#### 2.4.1. Quantitative Data

The Jamovi and JASP 2021 software were used to conduct these analyses. Paired-sample Student *t*-tests were run to assess evolution between students’ 21st competencies level before and after the program with effect sizes calculated through Cohen’s Delta as follows: a positive pre–post test score difference implies a reduction between the two respective scores, whereas a negative value will imply that the post-test score has increased on average as compared to the pre-test score. The quantitative data analysis included a range of exploratory analyses, such as correlation analyses, linear regressions, one-way ANOVA, MANOVA and descriptive analyses. It was hypothesized that factors such as age, gender, grade, country of residence and socio-economic level of the household might have effects that could be examined through these analyses. These exploratory analyses were not intended to address our hypotheses directly, but rather to offer supplementary insights to inform the discussion and guide future research.

Questionnaires were presented at the following two times: before and after the program ended. Pre-test questionnaires were presented and completed before starting the first individual session. Post-test questionnaires were carried out once the program was finished, after the last session. Students with high social desirability scores (i.e., with a score greater than 1.5 standard deviations from the mean, i.e., in this sample a score of 13) were excluded from analyses.

#### 2.4.2. Qualitative Data

Qualitative analyses were conducted on the reflective questions (RQA). As a reminder, these RQAs are proposed at the end of each session and were created to measure if the student proposes a response whose elements show that he/she has developed the sub-competency worked on in the session. A thematic analysis is conducted on each of the twelve reflective questions. A thematic analysis is a widely used qualitative method that consists of identifying, analysing and reporting “themes”, i.e., responses or meaning patterns within a verbatim data set (Braun and Clarke 2006; Terry et al. 2017). For this purpose, themes and sub-themes (i.e., here, implicit or explicit mention of the competency based on the sub-competency indicators) are identified. Themes have been subject to a collective procedure of reliability to avoid low inter-evaluator agreement and followed the 6-step process recommended by Braun and Clarke (2006). The construction of the themes in accordance with the thematic analysis methodology is based on the original CCR model (Fadel et al. 2015).

The thematic analysis was carried out using Tropes and Nvivo software. To categorize data, codes were made based on the self-reported acquisition of competencies and theoretical knowledge expressed by the student.

Initial data were visually represented through word clouds to ascertain the frequency of keywords and takeaways.

Preliminary coding followed an inductive approach that aimed at reducing and categorising. All answers from any student that participated, irrespective of their completion of the course, have been used. In fact, the reflexive questions can be analysed independently, in our opinion. Having a large number of respondents would increase the precision of our conclusions, and their potential generalisability, which is why we made this choice. Nevertheless, this has the disadvantage in our analyses of unbalancing the content of certain competencies in favour of others, especially those presented at the beginning of the program. Indeed, the number of participants is greater at the beginning than at the end. It is with these limitations in mind that the analyses are made.

#### 2.4.3. Triangulation

Finally, we crossed data from quantitative and qualitative data to see which 21st socio-emotional competencies were actually developed during this program. Triangulation consists of crossing data from different methodological approaches to study a phenomenon, a method that increases the validity and the understanding of the phenomenon studied through a deeper study of the data (Bekhet and Zauszniewski 2012; Sarwanto et al. 2021). In this research, data from a validated self-reported questionnaire (CCI, quantitative approach) were compared with data from a self-assessment competency inventory (exploratory quantitative approach) and data from self-reflection questions (qualitative approach) and matched an across-method triangulation as defined by previous authors (cf. Bekhet and Zauszniewski 2012; Risjord et al. 2001).

## 3. Results

### 3.1. Quantitative Data: CCI-21 and CBI Questionnaires

#### 3.1.1. Comparison between Pre–Post Average Scores CCI21

**Figure 2:**
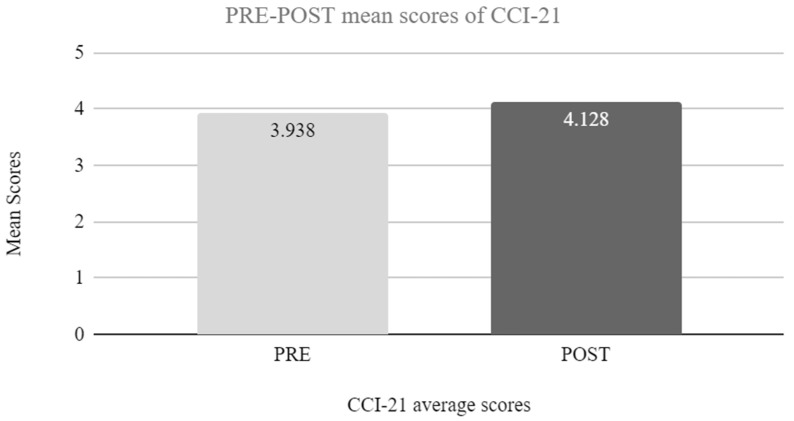
PRE–POST CCI21 average scores comparison.

Analysis showed that for average CCI-21 scores, there was a significant difference between post and pre-scores, [*t*_(23)_ = −2.656, *p* = 0.014, *d* = −0.542]. Participants showed better CCI-21 general average scores at the end of the program (*M =* 4.128, *SD =* 0.506) than at the beginning (*M =* 3.938, *SD =* 0.527, for details see Figure 2).

#### 3.1.2. Pre–Post Detailed Results Per CCI21 Competencies

We expected for each competency a statistically significant positive difference between the mean score obtained by our sample before and after the program. The following four out of the twelve 21st century competencies were specifically targeted by the BE Organized program: *Critical Thinking (CRI)*, *Metacognition (MET)*, *Mindfulness (MIN)* and *Resilience (RES)*. A paired-sample Student t-test analysis was carried out to look for significant intra-individual differences.

**Figure 3:**
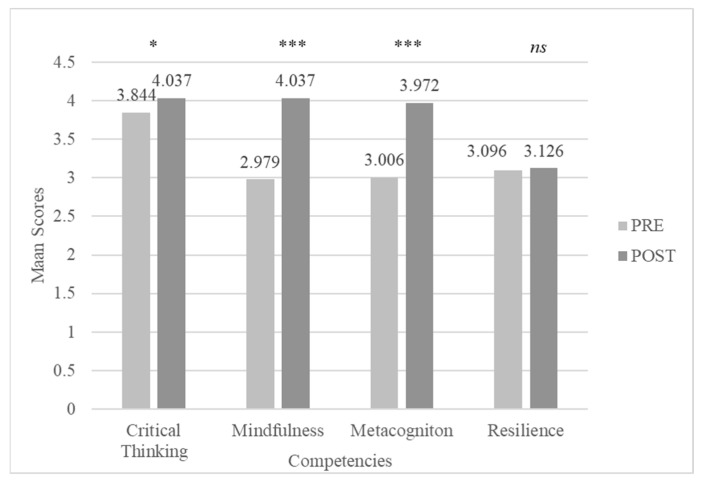
PRE—POST CCI21 targeted competencies scores comparisons. Note. ns: non-significant differences, *: *p* < .05, ***: *p* < .001.

Data show that three (i.e., Critical Thinking, Metacognition and Mindfulness) of the four main competencies targeted by the program were actually developed. Indeed, Critical Thinking has a post-program score (*M =* 4.037, *SD =* 0.699) that is significantly higher (*t*(23) = −2.243, *p* = .035, *d* = −0.458) than the pre-program score (*M =* 3.844, *SD =* 0.744). In the same way, participants scored significantly higher (*t*(23) = −8.416, *p* < .001, *d* = −1.718) on Metacognition at the end of program (*M =* 3.972, *SD =* 0.688) than at the beginning (*M =* 3.006, *SD =* 0.604). We found similar results for Mindfulness too (*t*(25) = −7.845, *p* < .001, *d* = −1.601), which is to say the highest post-program score (*M =* 4.037, *SD =* 0.575) than the pre-program one (*M =* 2.972, *SD =* 0.578). Yet, for Resilience, the post-program score (*M =* 3.126, *SD =* 0.593) is not significantly higher (*t*(23) = −0.697, *p* = .493, *d* = −0.145) than the pre-program score (*M =* 3.096 *SD =* 0.562, for details see Figure 3).

**Figure 4:**
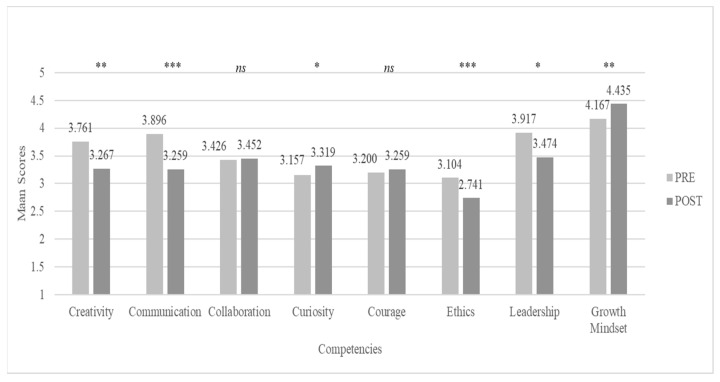
PRE—POST CCI21 non-targeted competencies scores comparisons. Note. ns: non-significant differences, *: *p* < .05, **: *p* < .01, ***: *p* < .001.

Although only one of the competencies targeted by the program seems to not have been significantly developed, analysis shows that there are significant differences between post and pre-program scores in six out of the eight other 21st century competencies, the following two skills: *Creativity* and *Communication*; the following three characteristics: *Curiosity*, *Ethics* and *Leadership*; for the other meta-learning: *Growth Mindset* (for details see Figure 4).

#### 3.1.3. Comparing Individual vs. Collective Session Developed Competencies (CBI)

The Competencies CheckBox Inventory (CBI) was used to self-report competencies developed during individual sessions as well as during group work in the collective sessions (Action Lab). A descriptive approach involving the frequencies of selections (or votes) made by the students has been used for analysis.

**Figure 5:**
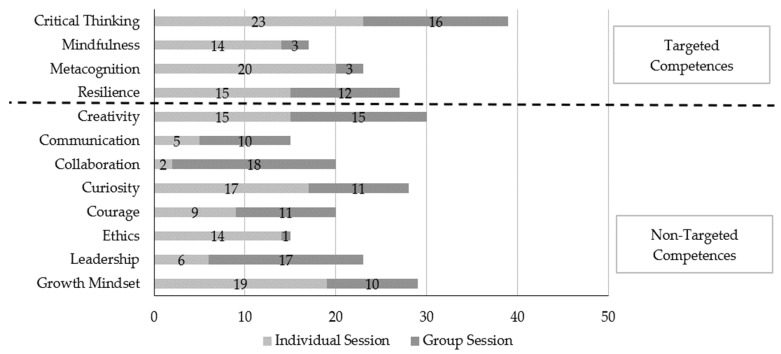
Self-rated evaluation of 21st century competencies developed during the program.

The general aim of the BE Organized program is to develop 21st century competencies. Specifically, the *individual sessions* propose a focus on the following 4 of the 12 competencies: *Critical Thinking*, *Metacognition*, *Mindfulness* and *Resilience*. We have therefore plotted in this graph (Figure 5) the number of students out of the 27 in the sample who identified one of these twelve competencies as being among the three developed in the individual sessions, and then the same question for the group sessions. For example, ethics was mentioned by 14 students in their top 3 skills developed by the individual sessions and mentioned by only one student for the group sessions. For the individual sessions, students frequently reported the development of Critical Thinking (23 votes), Mindfulness (20 votes) and Growth mindset (19 votes) as their top 3 most-developed competencies, 2 out of which, i.e., Critical Thinking and Mindfulness, fall under the targeted competencies. Additionally, in the Group work (known as Action lab by the participants), students were expected to develop Collaboration. Here, students self-reported the development of Collaboration (18 votes), Leadership (17 votes) and Critical Thinking (16 votes). Overall, Critical Thinking received the highest votes (39 votes) when combining both Individual and Action lab self-reported competency checkbox inventory results, then *Creativity* and *Growth Mindset*.

### 3.2. Qualitative Data

Reflective question assessments were analysed using a three-stage process; categorisation of student responses, two-fold competency mapping, interaction of target and observed competencies.

#### 3.2.1. Stage 1: Categorisation of Student Responses

The preliminary inductive coding resulted in eight themes/categories. The eight themes and the number of references that have been coded are explained below. They will be presented from the most referenced category to the least referenced.

1. *Session Description (411 References)*
  This category entails adjectives used to describe the session, and is further divided into the following subcategories:
  - Positive: Words such as “thought-provoking”, “fun” or “creative” fall under this sub-category;
  - Neutral: Words such as “Straight forward” fall under this category;
  - Negative: Words such as “Confusing” and “Boring” fall under this category.

2. *Interacting with Decisions (384 References)*
  - Adapting Flexibly: “Life is uncertain and we need to expect the unexpected and there are rigid and flexible reactions where flexibility is better.”;
  - Consideration of different perspectives: “As we are acknowledging different perspectives of the outcome, this would affect the end result. We are also reflecting and reviewing the surroundings and analysing every detail possible as with logical thinking—a tiny mistake could rapidly cause danger.”;
  - Critical Thinking and Problem Solving: “By thinking reasonably, we can logically process information and situations, this allows us to find answers and solve problems easily, it also makes us judge situations carefully.”;
  - Game theory: “Being able to identify games that are finite and infinite”;
  - Logical Reasoning: This is further divided into subcategories: Awareness of Biases, Awareness of Consequences, Cognitive Dissonance, Recognising and Regulating emotions and Value orientation;
    ○ Awareness of Biases: “Discrediting, Emotional manipulation, trolling, polarization, impersonation, conspiracy/So you don’t fall into one of these cognitive biases, and so you don’t start spreading misinformation about people.”;
    ○ Awareness of Consequences: “Both positive and negative outcomes of the decision are identified using reasoning. If one is very upset with another person, reasoning may suggest hitting the other person in the face, but it also warns about the potential consequences.”;
    ○ Cognitive Dissonance: Being aware of cognitive dissonance helps you absorb the facts that differ from what you believe;
    ○ Recognising and Regulating Emotions: Your emotions and moods, especially if you are in a leading role affects the emotions, moods and attitudes of the people you lead or around you. To ensure that you are ok and have good emotions and thoughts because they can reflect on the people around you;
    ○ Value Orientation: “Whenever I have to make a decision, firstly I have to base it on my values because in the long term it can affect not only me but others.”

3. *Interacting with Information (350 References)*

Includes all responses related to acquiring, organizing, processing, critiquing and transferring of information.

- Assessing Information Reliability: This sub-category is further broken down into the Need for Information assessment and Strategies for Information assessment;
  ○ Need for Information assessment: This category is further broken down into Disinformation and Misinformation and Fake news;
    ▪ Disinformation and Misinformation: “Misinformation-False information, not necessarily to harm someone. Disinformation-False information posted purposely to harm someone. I think it is important to assess the quality of information I receive because then I know that everything I am referring to is true;
    ▪ Fake News: “Be careful with information online, some of it might be fake.”
  ○ Strategies for Information assessment: This category is further broken down into the 5Ws, Conscious Consumption Strategies and CRAAP;
    ▪ 5Ws: “Using the 5 whys strategy can help you reflect on your thinking process.”;
    ▪ Conscious Consumption Strategies: “Check the source of the information, compare information with facts, in case of breaking news wait until more information is available…”;
    ▪ CRAAP: “Assessing information in terms of reliability can be done using the CRAAP strategy”.
  - Curiosity and Research Skills: “Curiosity is crucial for flexibility, to keep an open mind and to not stop seeking opportunities.”;
  - Information Assimilation and Literacy: “If information is processed and assimilated through literacy skills, it enables us to determine and classify the usable, pertinent and precise knowledge and therefore organizes the basis of knowledge”;
  - Information Overload and Anxiety: “I would sum up this session as a presentation where I was able to learn and see examples of information anxiety and information overload.”;
  - Information Management, Organization and Processing: This sub-category is further divided into the Information Processing and Organization, and Need for Information Management, Organization and Processing:
    ○ Need for Information Management, Organization and Processing: “Importance of data processing includes increased productivity and better decisions”;
    ○ Information Processing and Organization: It is further divided into Keywords, LATCH technique, Mind Map, Simplify and Summarize;
  - Self-Assessment and Tests for Understanding: “I learnt how I can make concept maps to gather information and self-assess my own work!”

4. *Personal Wellbeing and Development (84 References)*
  Personal Wellbeing and Development is an overarching category that includes strategies and mindsets required to be mentally healthy and driven.
  - Character Strengthening: “Because it strengthens your character”;
  - Gratitude: “Don’t forget to say a simple thank you.“;
  - Growth Mindset: “It is important as it helps our minds to grow and open up to new things.”;
  - Mindfulness and Internal regulation: “It is important to regularly monitor your internal state because it can help you control your thoughts and if you do not do this then it could have a negative impact on you and other people without even knowing it.”;
  - Seeking Help: “I should get to know when to stop struggling and not be ashamed to ask for help.”;
  - Strength and Weakness: “It (the session) helped me to realize my strength and weakness in order to enhance and improve the quality of my piece of work.”;
  - Stress Reduction and Managing Screen Time: “SNS (Social Networking Sites) and other digital platforms can cause stress. To deal with stress, take a deep breath or take a break!”

5. *Exploring daily application or future benefits (64 References)*

This category entails sentences used by students to declare that the session will help them daily or in the future. Some sentences dig deeper into how *exactly* the session will help them. “It will help me get my perfect career” and “This session was important for the problems we face everyday.”

6. *Interacting with Goals (62 References)*
  This category further breaks down the process of goal setting and its attainment. The subcategories include the following:
  - Goal Achievement: “It’s a good technique to achieve our goals… it can remind me of my goal and motivate me!”;
  - Monitoring Progress: “It’s easy to set a goal but hard to keep on it when no feedback is available. Monitoring might help me see flaws in my strategy”;
  - SMART Goals: “S: Specific, M: Measurable, A: Attainable, R: Relevant, T: Time-bound”. Breaking down goals using the SMART framework leads your goals to be more clear and reachable”.

7. *Reflective Practices (60 References)*

Includes all excerpts from student responses that share the importance of and have claimed to practice reflection during the session. “This session made me really think if I had made good or bad decisions in my life.” “In order to structure our lives, we need to justify and reflect on our values and decisions.”.

8. *Motivation and Resilience (11 References)*

Under this category, all excerpts that mention the importance of and the session’s focus on Motivation and Resilience are included as follows: “Seeing my achievements motivates me to continue and never give up because of how far I’ve gone”.

#### 3.2.2. Stage 2: Two-Fold Competency Mapping

In the second stage, the eight categories and their corresponding subcategories were studied and mapped to specific competencies and sub-competencies (for details see Table 2). This is referred to as observed competencies. The eight categories and their subcategories were therefore mapped into *Observed Critical Thinking*, *Observed Resilience*, *Observed Metacognition*, *Observed Mindfulness and Others*. Secondly, each session was also mapped to the competency it intended to develop. This is known as Targeted competency.

Secondly, each session was also mapped to the competency it intended to develop. This is known as Targeted competency (see Table 2 Above). Based on this reorganization of data, we can cross those observed competencies and targeted competency of sessions.

#### 3.2.3. Stage 3: Interaction of Target and Observed Competencies

At stage 3, the intersection of what is targeted and what is observed between the sessions is studied.

The Table 3 show the percentage of coding (coverage) that is associated with Observed and Target competency. When the percentage of codes that observe a certain competency is relatively higher in the target sessions of the same competency, then an assumption that the target session achieved its goal is made.

At first glance, it is evident that most coverage of codes is seen within the Target competency of Critical Thinking. Since Critical Thinking sessions are the first sessions of the BE Organized program (sessions 1, 2, 3 and 4), the number of participants was higher than in the subsequent sessions. Mindfulness has the least number of codes because it entails only two sessions (as opposed to 4 critical thinking sessions), and the answers were shorter, owing to technical errors in the BE platform. To compare observed competencies within a target, it is, therefore, more useful to take a rank-based intra-target percentage approach than a global approach. For instance, consider “Observed Mindfulness” in sessions targeting Critical Thinking vs. sessions targeting Metacognition. Although targeted, Critical Thinking sessions have a higher percentage of Observed Mindfulness (42.01%), it is ranked 3rd among the Observed Competencies in the isolated target of Critical Thinking. Here, it is ranked after Critical Thinking (77%) and Metacognition (77%). This is different in the case of targeted Metacognition sessions, where Observed Mindfulness (23.82%)—albeit a lower percentage than that found in Critical Thinking—is ranked first among the observed competencies, followed by Metacognition (15.23%) and Critical Thinking (14.90%).

Finally, we could have hypothesized that the order of the sessions has an impact on the level of recollection (Mindfulness at 4.74% appears before Metacognition, which has a higher rate of 15.23%). However, upon examining the table, it becomes evident that this is not the case. Instead, it appears that the targeted objectives for character traits, specifically Mindfulness and Resilience, were not achieved, as indicated by the recovery percentages below 10%. This observation raises questions regarding the adequacy of the proposed content in reinforcing these competencies. i.e., we could make the hypothesis that traits, skills and meta-learning, even if they all belong to the 21st century competencies, could not be developed online in the same way, i.e., we could make the hypothesis that traits, skills and meta-learning, even if they all belong to the 21st century competencies, could not be developed online in the same way.

We proceed to a supplementary analysis by doing a radar chart that shows the compositions of coverage of codes within target competencies on the session, and they are available as an appendix with a descriptive analysis for better readability (see Appendix C).

### 3.3. Triangulation

Critical Thinking, Mindfulness, Metacognition and Resilience are 4 of the 12 Competencies that have been targeted in the Beyond Education BE-Organized program. The acquisition or development of these competencies can be studied through the following different data sources: CCI-21, RQAs and CBI. As data are drawn from multiple sources, it broadens the researcher’s insight into the different issues underlying the phenomena being studied (Bekhet and Zauszniewski 2012). At this stage, each competency will be considered, and its performance in terms of each instrument/indicator will be studied (see Table 4).

#### 3.3.1. Critical Thinking

CCI-21: Critical Thinking presents a post-program score that is significantly higher than the pre-program score.

CBI: Overall, Critical Thinking has received the highest votes (39 votes) when combining both Individual and Action lab self-reported competency checkbox inventory results.

RQA: Critical Thinking (72.70%) holds the first ranking among the four observed competencies within the target sessions. This indicates that there is an overlap between what is observed and what is targeted, thereby suggesting that the sessions have indeed been effective in helping students attain their objectives as follows: showing different expressions of the sub-competency that underlies the session to work on this competency. Additionally, owing to its strategic location at the beginning of the course, it received a global majority of responses.

#### 3.3.2. Resilience

CCI-21: In terms of Resilience, the post-program score is not significantly higher than the pre-program score.

CBI: While considering Individual session votes in the Checkbox inventory, Resilience, tied with Creativity, received the fourth highest votes (15) after Critical Thinking, Growth mindset and Curiosity. Additionally, it received 12 votes in terms of its development in group sessions, and in total, it received 27 votes, which is the fifth-highest score. The individual sessions targeted four competencies, and Resilience lies within the top four competencies that seem to have been developed according to student votes.

RQA: Resilience (66.59%) holds the first ranking among the four observed competencies within the target sessions. This indicates that there is an overlap between what is observed and what is targeted, thereby suggesting that the sessions have indeed been effective in attaining their objectives.

#### 3.3.3. Mindfulness

CCI-21: There is a positive difference between the post-program score and the pre-program score for Mindfulness.

CBI: Mindfulness received the second highest votes (20) in the Individual session category and three votes in the group category, with a total of 23 votes. The individual sessions targeted four competencies, and Mindfulness lies within the top four competencies that seem to have been developed according to student votes.

RQA: Only 4.74% of responses that explicitly or implicitly express the development of mindfulness (Observed Mindfulness) fall within the Target Mindfulness sessions. However, there seems to be a more equitable distribution of Mindfulness across all sessions, with the most observations being under Critical Thinking target sessions (42.01%).

#### 3.3.4. Metacognition

CCI-21: Participants scored significantly higher on Metacognition at the end of the program than at the beginning.

CBI: Metacognition received a total of 17 votes. In the individual session, 14 students believed that the Metacognition was among the top 3 competencies developed and only 3 students believed that Metacognitions was one of the top 3 competencies developed in group sessions.

RQA: Only 15.23% of observed “metacognitive” responses coincide with sessions that target metacognition. Observed Metacognition is ranked second after Mindfulness (23.82%).

## 4. Discussion

### 4.1. Summary

BE Organized aimed to develop the following four targeted competencies during online asynchronous individual sessions: *Critical Thinking*, *Metacognition*, *Mindfulness* and *Resilience*. The three methodological approaches (CCI-21, self-report questionnaire [quantitative]; CBI: self-evaluation of the twelve 21st century competencies [quantitative]; RQA, reflective questions on each of the 12 sub-competencies targeted [qualitative] taken together allow us to say that the four competencies targeted have been developed, as at least two methods agreed on that. When the four targeted competencies are taken separately, it is not the case. In addition, some other 21st century competencies seem to have been impacted by the program, meaning that they either increased or decreased after the program, notably *Creativity*. Indeed, this competency decreased after the program ended. The Xs mean that this competency has been impacted, in any direction, by the program (see Table 5).

Indeed, except for Critical Thinking, for which all three methodological approaches agree, the other three competencies are developed depending on the approach adopted. We will see why some of these competencies might not have been developed.

Results based on a quantitative approach show that the last one was not significantly developed. We can make the hypothesis that *Resilience* was overshadowed by other main competencies or that this competency needs more sessions to be significantly developed (Fazey et al. 2007). By definition, Resilience requires a high level of adaptability on the part of individuals (Fadel et al. 2015). In studies conducted on Resilience, to become adaptive, individuals have to learn and develop variation in their expression of abilities and skills (Fazey et al. 2007). Moreover, resiliency is obtained through multiple challenging episodes of life, for example, in the academic environment, which students face and must persevere (Tough 2016). Research suggests that Resilience is built over time (Ungar 2015). Indeed, it takes time to put in place these various practices and to think them through because of the transferability of a practice from one situation to another; in short, becoming an expert cannot be performed without a meta-learning faculty (Fazey et al. 2007).

Secondly, CBI results on individual 21st century competencies show that *Critical Thinking* and *Mindfulness* are judged by the students as being developed, but this is less the case for Metacognition and *Resilience*. *Resilience* has already been discussed. Indeed, Metacognition is significantly developed when results are analysed with the questionnaire perspective (CCI-21), whereas with self-evaluation (CBI) is not. It may be that to self-evaluate, metacognition can be hard for a student of their age. In general, it is more difficult for students to verbalize their metacognition (Bosch et al. 2021) since metacognition is not a competency that is traditionally taught in educational systems (Crippen and Antonenko 2018; Joseph 2009). Yet recent research has shown that verbalizing metacognition in students can lead to better-computerized learning and the development of metacognitive skills (Bosch et al. 2021).

Thirdly, results on RQAs show that *Mindfulness* might not have been developed. The lack of development of *Mindfulness* observed in the RQAs may be a result of one of the two sessions aimed at developing this competency the RQA implemented was not the right one (while we were treating the data, we discovered that the previous session’s RQA—targeting Metacognition—was integrated instead). So only one question out of twelve questioned students’ Mindfulness, which seems not enough compared to the *Critical Thinking* competency, which had four questions measuring it. In addition, when the only RQAs on *Mindfulness* are analysed, a possible confusion between *Metacognition* and *Mindfulness* is seen. Moreover, some research has shown a certain degree of conceptual overlap between these two concepts. Indeed, the awareness component was found in an empirical study to be common to both competencies (Mörck 2009). Moreover, we surmise that the latter competency (Mindfulness) presupposes the action of the former (metacognition). Thus, there may be confusion for students and the need to make the specificity of each of these competencies more explicit in future programs. In addition, Mindfulness seems to have been worked across all sessions, meaning that this competency might be needed to develop all of them or that the session needs to be redesigned to make them more explicit in their objective to develop *Mindfulness*.

The results found—on the basis of the CCI and CBI, the RQAs only measuring competencies other than those targeted—also showed the possibility that competencies other than those targeted have been developed. We will discuss some of them, notably *Creativity* and *Growth Mindset*. Several explanations can be envisaged, first by considering the two competencies simultaneously and then in relation to the objective of the program. Indeed, it might not be surprising that an online program aimed at self-improvement is developing students’ *Growth Mindset* and *Creativity*. In general, the completion of this type of educational program leads by “definition” to a progression for young people in their *Creativity* and *Growth mindset* competencies, progress at school and at work and in life (Lipnevich et al. 2016; Poropat 2009). It is true that we can consider that the BE Organized program is about overseeing one’s life, which implies, in a more or less secondary way, to be the “master of one’s own life,” and in a position as the leader of oneself. To be a good leader of one’s own life requires us to learn every day about ourselves, to have a “Growth Mindset”, and to be curious about what surrounds us. Moreover, for things to be performed, they must be performed well. Therefore, for the behaviour and actions of individuals to be optimal, they should adopt ethical practices. The six competencies developed could make sense when we understand BE Organized this way. Finally, participation in the group sessions could also allow participants to develop these competencies. When we cross CCI-21 with the CBI, we can see that there are also some of them, particularly *Creativity*, developed during group sessions. Growth Mindset and Creativity have been shown to be developed within extracurricular activities such as BE Organized (Narkabilova and Khujamberdiyeva 2021; McClendon et al. 2017). It is then not so surprising to find them developed here. However, these results are only based on the student’s perceptions. In the future, we will explore more systematic ways to assess 21st century competencies that are specifically developed during group sessions. It is always difficult to assess these competencies within a group without using a long and cumbersome process (e.g., Zhuang et al. 2008).

### 4.2. Limits and Further Research

Limitations that underlie this research work include the effect size and lack of statistical power. Indeed, we have a very small sample of students, so the conclusions cannot be easily generalized. Post-hoc analyses made with G*power and conducted with a sample of 27 and a repeated measures *t*-test, with a significance level at *p* < .05 and a pre–post difference of 0.5 points on average, indicate a power slightly higher than 0.80, which is quite low. Thus, all the conclusions should be interpreted carefully. In addition, even if we assume that we limited desirability bias by making students complete the SDS scale (Crowne and Marlowe 1960), we still need to recognize that self-assessment produces such an effect, even if we do our best to limit it. Indeed, as we are using the same methodology (i.e., self-report questionnaire) to reduce this desirability, we cannot say with confidence that this bias is completely tackled. Yet, some authors have also clearly shown that self-assessments are reliable as they produce consistent results over time across tasks or in case items (Ross 2006). Therefore, the results obtained must serve as a basis for further work and reflection. Nevertheless, this work can allow us to make recommendations, notably because we have adopted a mixed method/triangulation approach (Bekhet and Zauszniewski 2012). By crossing approaches, we can make conclusions with more “certainty” than with a single methodological approach.

In addition, the analysis conducted here might be improved. These exploratory data could have been beneficial from additional analyses, for which the software used are not adapted but especially for which a larger sample size would be more appropriate. The paired sample *t*-test could have been adjusted by a Dunn–Sidak correction (correcting for the accumulation of alphas), which does not require independent hypothesis testing since the competencies would not be completely independent of each other (Midway et al. 2020). In the same way, contrasting the scores obtained between the competencies targeted and those not targeted would be a new way of analysing the results. Indeed, as these competencies can be thought of as a whole, a set, it would be interesting if the students are more “organized”, i.e., if they have developed the four targeted competencies simultaneously (group 1) compared to the eight non-targeted competencies (group 2), and not simply to consider whether each of them is “independently” developed.

If it is true that research favours randomized experimental designs (Midway et al. 2020), there is also a growing preference for more ecological designs, leading many to favour alternative quasi-experimental models (Midway et al. 2020). However, the question of internal validity remains, and of inferential power, the author advises that to improve these points, it would be necessary to have more stable associations, to turn to replication, to have several control groups, to consider non-equivalent dependent variables or even more points of pre-test observations (Midway et al. 2020).

Taking into account these previous recommendations, the development of this program has been informed by the insights obtained from two prior programs. The feedback received regarding the content and structure of those programs has been integrated to enhance the effectiveness of the current program. Additionally, before the program was launched, several focus groups involving students were conducted. Allowing for student inputs, and given that the students’ belief that the program helps them, producing a positive effect for the program- this could lead to an increase in internal validity (Anastasi 1988; Bornstein 1996). Nevertheless, future studies should turn to the concept of non-equivalent dependent variable, considering that the competencies that are not targeted belong to this group.

However, a question arises regarding the non-equivalence of certain non-valued competencies when compared to others, particularly in relation to group sessions and the extent of the connections established between a non-valued competency and one or more non-valued competencies. For instance, creativity, which is considered non-valued, theoretically has connections with other competencies such as Creative Thinking (OECD 2019) and Mindfulness. However, the link between courage and criterion-referenced thinking is not consistently investigated or clearly defined within the field.

Certain competencies are developed whether or not they are explicitly mentioned (cf. results from the pre–post comparison of CCI scores that are presented in the Results Section 3.1.2—pre–post detailed results per CCI21 competencies—or for details see Table 2), notably creativity, which is not surprising when we know that in the model, creativity is a skill, which refers to “how individuals use that knowledge”. Creativity, and other skills (namely, Critical Thinking, Communication and Collaboration), therefore, might be essential to the development and use of the other competencies at least that is what these first results suggest. However, contrary to what one might expect, it is important to note that the level of creativity has generally decreased over the past 20 years (Kim 2011). Moreover, an overly conforming environment can have a dampening effect on creativity (Beghetto 2017; Kim 2011). Sometimes, there is a tendency to underestimate the freedom individuals or students have to think and act in unique and non-routine ways (Beghetto 2017). In the context of this program, the content of the Mindfulness sessions focused on developing habits and routines. As a result, it can be hypothesized that at this stage of learning, students might be encouraged to be less creative. However, this stage could be essential for them to express their creativity based on the knowledge they have acquired. Further analysis, particularly of the responses to the RQA, can be conducted to explore whether the competencies identified as being impacted in a negative direction by the CCI-21 are indicated in students’ RQA answers.

These results must be qualified in view of the small sample size, but they provide a good basis for work and reflection on the creation of effective psychosocial competency development programs. First, the importance of targeting specific competencies and limiting the number worked on in a program. We can see that Critical Thinking stands out among the other four competencies in a very clear way, whereas four seems a priori a small number of competencies targeted.

Finally, an evidence-based approach seems to be a guarantee for the effective development of 21st century competencies, which requires building programs on theoretical models derived from research by defining pedagogical objectives and measurement indicators in accordance with the chosen model.

## 5. Conclusions

This study hypothesized that students would have significantly improved competencies such as *Mindfulness*, *Critical Thinking*, *Metacognition* and *Resilience* after completing the BE Organized course by Beyond Education. The triangulation of data sources investigated the BE Organized program using different lenses—both qualitative and quantitative. It also shifted its focus between the global impact of the program and the session-specific impact. The first preliminary results show a definitive improvement in the *Critical Thinking* competency, owing to significant results extracted from all three data sources. The improvement of the other three competencies varies with different data sources. This could be a result of the following numerous factors: better instructions and/or session structure in *Critical Thinking* sessions, Strategic positioning of *Critical Thinking*—as Critical Thinking sessions were the first presented to students, or simply the popularity of the word itself. In the future, to improve and test those hypotheses, we can evaluate session impact individually (not within a program) or to make the objectives of the sessions (i.e., the development of the sub-competency of the targeted competency) clearer and more explicit. These results must be qualified in view of the small sample size, but they provide a good basis for work and reflection on the creation of effective 21st Century socioemotional competency development programs. Finally, certain competencies are impacted whether or not they are explicitly mentioned, notably *Creativity*. Indeed, *Creativity* is a skill, meaning, in the four-dimensional model (Fadel et al. 2015), which is key to being able to express any of the following competencies in our program BE Organized: *Critical Thinking*, *Metacognition, Mindfulness* and *Resilience* and maybe in any on- or off-line programs that aim to develop 21st century competencies.

## Figures and Tables

**Table 1:** Target competency of sessions.

Session (S)	Target Competency
S1.	Critical Thinking
S2.	Critical Thinking
S3.	Critical Thinking
S4.	Critical Thinking
S5.	Mindfulness
S6.	Mindfulness
S7.	Mindfulness
S8.	Metacognition
S9.	Metacognition
S10.	Metacognition
S11.	Resilience
S12.	Resilience

**Table 2:** Crossing qualitative coded categories and observed competencies.

Categories	Sub-Categories	Observed Critical Thinking	Observed Mindfulness	Observed Metacognition	Observed Resilience	Observed Others
Session Description	Positive description					X
	Neutral description					X
	Negative description					X
	Adapting Flexibly			X		
	Consideration of different perspectives	X				
Interacting with Decisions	Critical thinking and Problem Solving					
	Game theory			X		
	Logical Reasoning	X				
	Assessing Information reliability	X				
	Curiosity and Research Skills					
	Information Assimilation and Literacy	X				
Interacting with Information	Information Overload and Anxiety				X	
	Information Management, Organization and Processing			X		
	Self-Assessment and Tests for Understanding			X		
	Character Strengthening				X	
	Gratitude					
	Growth Mindset				X	
Personal Wellbeing and Development	Mindfulness and Internal regulation		X			
	Seeking Help				X	
	Strength and Weakness				X	
	Stress Reduction and Managing Screen Time		X			
Exploring daily application or future benefits						X
Interacting with Goals	Goal Achievement					
	Monitoring Progress			X		
	SMART Goals			X		
Reflective Practices				X		
Motivation and Resilience			X			

**Table 3:** Percentage of intersection between Target and Observed competency.

		Observed Competency			
		1.	2.	3.	4.
Target competency	1. Critical Thinking	72.70%	42.01%	72.22%	23.54%
	2. Mindfulness	9.19%	4.74%	11.88%	1.36%
	3. Metacognition	14.90%	23.82%	15.23%	66.59%
	4. Resilience	3.20%	29.42%	0.67%	8.50%

**Table 4:** Data triangulation checkbox of targeted competencies.

	CCI-21	CBI	RQA
Critical Thinking	X	X	X
Mindfulness	X	X	
Metacognition	X		
Resilience		X	X

**Table 5:** Data triangulation checkbox of all competencies.

Targeted	CCI	CBI	RQA	Non-Targeted	CCI	CBI	RQA
Critical Thinking	X	X	X	Creativity	X	X	Not measured
Metacognition	X	X		Communication	X	X	Not measured
Mindfulness	X	X		Collaboration		X	Not measured
Resilience		X	X	Curiosity	X	X	Not measured
				Courage		X	Not measured
				Ethics	X	X	Not measured
				Leadership	X	X	Not measured
				Growth Mindset	X	X	Not measured

**Figure A1:**
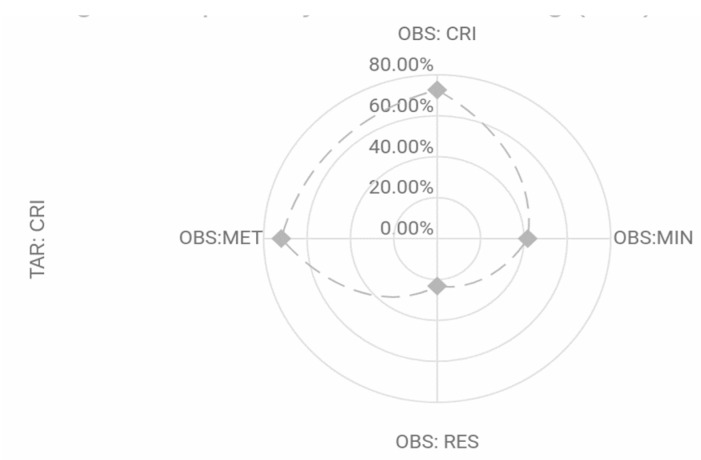
Radar chart showing the composition of coverage of codes within Targeted Critical Thinking session.

**Figure A2:**
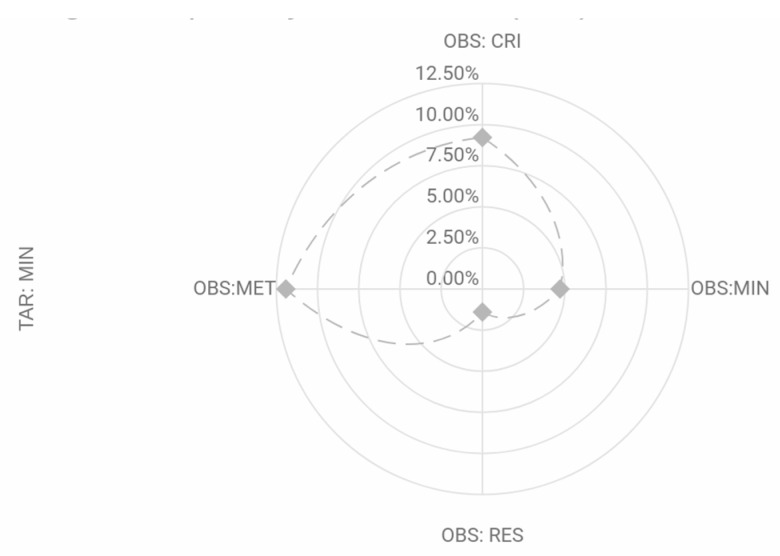
Radar chart showing the composition of coverage of codes within Targeted Mindfulness session.

**Figure A3:**
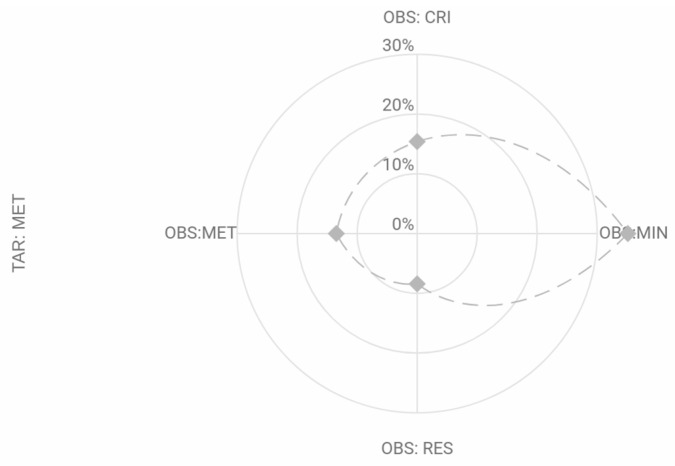
Radar chart showing the composition of coverage of codes within Targeted Metacognition session.

**Figure A4:**
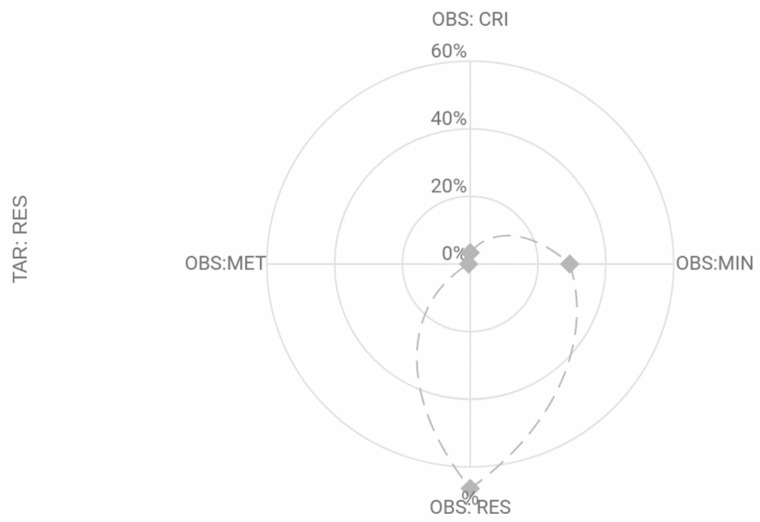
Radar chart showing the composition of coverage of codes within Targeted Resilience session.

## Data Availability

Data are available on request.

## Appendix A. Competency Checkbox Inventory (CBI)

Part 1—Could you select the top three competencies you believe you have developed in the individual sessions:

- Critical Thinking
- Creativity
- Communication
- Collaboration
- Mindfulness
- Curiosity
- Courage
- Resilience
- Ethics
- Leadership
- Metacognition
- Growth Mindset

Part 2—Could you select the top three competencies you believe you have developed in the group sessions:

- Critical Thinking
- Creativity
- Communication
- Collaboration
- Mindfulness
- Curiosity
- Courage
- Resilience
- Ethics
- Leadership
- Metacognition
- Growth Mindset

## Appendix B. Session Specific Questions Asked in the Reflective Question Assessments (RQA)

**Session No.**	**Sub-Competency**	**Sub-Competency Specific Questions**
Critical Thinking		
1	CRI1: Identifying, clarifying and organizing information	Why is it important to process the information you receive?
		*n* = 93
2	CRI3: Applying sound reasoning to decision-making	How might logical reasoning impact our decision making?
		*n* = 74
3	CRI4: Assessing validity and quality of information	Why do you think it is important to assess the quality of information you receive?
		*n* = 67
4	CRI5: Reflecting critically on one’s own reasoning and assumptions	Why would it be important (or not) to be critical towards your own reasoning and opinions?
		*n* = 54
Mindfulness		
5	MIN2: Understanding by describing one’s emotions and reactions	Why might it be essential to reflect on one’s identity, experiences and achievements?
		*n* = 51
6	MIN3: Building effective habits for regulation of inner experience	How monitoring your progress might help you achieve your goals?
		*n* = 49
Metacognition		
7	MET1: Reflecting on processes, achievements, learning and/or identity	How monitoring your level of understanding might help you?
		*n* = 36
8	MET3: Monitoring comprehension and managing information accordingly	How useful is it to evaluate the consequences of your actions? Why?
		*n* = 35
9	MET4: Evaluating one’s actions and their consequences	Why is it important to regularly monitor your internal state?
		*n* = 35
Resilience		
10	RES1: Adapting flexibly	According to you: Which aspects of your life might improve if you start adapting flexibly?
		*n* = 33
11	RES3: Managing stress and expressing emotions appropriately	How not managing your emotions or stress might impact you? How it might impact others?
		*n* = 30
12	RES5: Persevering through challenges but seeking help when needed	When you are facing a challenge, how would you manage to determine when it is time to seek help?
		*n* = 29

## Appendix C. RQA Supplementary Analysis

1. Interaction of Target and Observed competencies: Critical Thinking

The chart shows that 72.70% of responses that are categorized under “Observed Critical Thinking”, are seen within the first four sessions that specifically target Critical Thinking. Under the Target Critical sessions, observed Critical Thinking holds the *first rank*. This indicates that students implicitly/explicitly expressed and demonstrated having learned critical thinking during these sessions. Additionally, 72.22% of responses that are categorized under “Observed Metacognition” (Rank II) are also seen in these four sessions that target Critical Thinking. Students’ responses in these sessions also show evidence of Mindfulness (42.01%, Rank III) and Resilience (23.54%, Rank IV).

2. Interaction of Target and Observed competencies: Mindfulness

The chart shows only 4.74% of responses that are categorized under “Observed Mindfulness” (Rank III) are seen within sessions 5 and 6, which specifically target Mindfulness. This indicates that not many students implicitly/explicitly expressed and demonstrated having learned Mindfulness during these sessions. However, 11.88% of responses that are categorized under “Observed Metacognition” (Rank I) and 9.19% of responses categorized under “Observed Critical Thinking” (Rank II) are seen in these 2 sessions that target Mindfulness. These sessions observe Resilience (1.36%, Rank IV) the least.

3. Interaction of Target and Observed competencies: Metacognition

The chart shows that 15.23% of responses that are categorized under “Observed Metacognition”, are seen within sessions 8, 9 and 10, which specifically target Metacognition (Rank II). This indicates that only some students implicitly/explicitly expressed and demonstrated having learned Metacognition during these sessions. Additionally, 23.82% of responses that are categorized under “Observed Mindfulness” (Rank I) and 15.23% of responses categorized under “Observed Critical Thinking” (Rank II) are seen in these 3 sessions that target Metacognition. These sessions do not observe a large extent of Resilience (8.50%) (Rank IV).

4. Interaction of Target and Observed competencies: Resilience

The chart shows that 66.59% of responses that are categorized under “Observed Resilience”, are seen within sessions 11 and 12, which specifically target Resilience. Under the Target Resilience sessions, Observed Resilience thinking holds the *first rank*. This indicates that students implicitly/explicitly expressed and demonstrated having learned Resilience during these sessions. Additionally, 29.42% of responses that are categorized under “Observed Mindfulness” (Rank II) are also seen in these 2 sessions that target Resilience. These sessions do not observe a large extent of Critical Thinking (3.20%, Rank III) and Metacognition (0.67%, Rank IV).
